# *Notes from the Field:* Seasonal Human Influenza A(H3N2) and Influenza A(H1N1)pdm09 Reassortant Infection — Idaho, 2019

**DOI:** 10.15585/mmwr.mm6914a4

**Published:** 2020-04-10

**Authors:** Randi Pedersen, Vonnita Barton, Jennifer Tripp, Lenee Blanton, John Barnes, Christine Hahn

**Affiliations:** ^1^Division of Public Health, Idaho Department of Health and Welfare; ^2^Southwest District Health, Caldwell, Idaho; ^3^Influenza Division, National Center for Immunization and Respiratory Diseases, CDC.

On February 17, 2019, a male patient aged 13 years with no underlying medical conditions was evaluated in an Idaho hospital emergency department for a 1-day history of fever (103°F [39.4°C]), dry cough, sore throat, headache, and weakness. A respiratory specimen was collected and tested positive for influenza A by rapid influenza diagnostic test (RIDT). The patient was treated with oseltamivir as an outpatient and recovered. As part of routine surveillance, a second specimen collected during the emergency department visit on February 17 was forwarded to the Idaho Bureau of Laboratories (IBL), where CDC’s influenza reverse transcription–polymerase chain reaction (RT-PCR) diagnostic panel detected both pandemic influenza A and H3, which suggested an influenza A(H3N2) variant virus of swine origin. The specimen was sent to CDC’s influenza diagnostic laboratory for confirmation, and the patient was interviewed.

During the week preceding illness onset, the patient did not travel and reported no animal exposure; he had not received a 2018–19 seasonal influenza vaccine. One household member developed respiratory symptoms on February 23, 2019, and sought care at an outpatient clinic, where a respiratory specimen tested positive for influenza A by RIDT. No specimen was available for additional testing, and no other exposures were identified. No additional household members reported respiratory symptoms.

Next generation sequencing at CDC revealed a new seasonal human influenza A(H3N2) and A(H1N1)pdm09 reassortant virus, rather than an influenza A(H3N2) variant virus of swine origin. Reassortment occurs when two influenza viruses infect a single host cell and exchange gene segments, creating a new virus. Sequencing data suggested that the patient was not coinfected and that the reassortment event likely occurred in another person. Phylogenetic analysis determined that the hemagglutinin genes belonged to human H3 subclade 3C.3a and neuraminidase genes belonged to human N2. Gene segments PB2, PB1, PA, NP, M, and NS displayed genetic similarity to human-origin influenza A(H1N1)pdm09 viruses. Genetic markers that would confer reduced susceptibility to oseltamivir, peramivir, and zanamivir were not detected. Viruses in H3 subclade 3C.3a react poorly by focus reduction assay with ferret antisera raised against A/Singapore/INFMH-16–0019/2016(3C.2a1), signifying that the 2018–19 Northern Hemisphere influenza vaccine* might not be protective against this virus.

As part of enhanced surveillance, the hospital where the patient sought care forwarded an additional 45 specimens that tested positive by RIDT for influenza A, collected during January 1–April 27, 2019, to IBL. Using the CDC influenza RT-PCR diagnostic panel, IBL determined that 23 (51.1%) were influenza A(H1N1)pdm09, 13 (28.9%) were influenza A(H3N2), and influenza was not detected in nine (20.0%) specimens. IBL sent 17 of the 45 specimens to CDC for sequencing; no additional reassortant viruses were identified.

At the time of the patient’s illness onset, influenza A(H1N1)pdm09 and A(H3) were cocirculating in Idaho (Figure), increasing the likelihood of coinfection and reassortment. Influenza A reassortment is observed at high rates in animal and cell culture models, but a biologically successful human reassortant virus is rarely reported (*1*–*3*). This is CDC’s first detection of this type of seasonal human influenza A(H3N2) and influenza A(H1N1)pdm09 reassortment. CDC recommends that state and local health departments, hospitals, and clinicians maintain surveillance to identify patients who might be transmitting newly emerging influenza viruses.^†^^,^^§^ CDC will continue virologic surveillance to monitor influenza genetic evolution and inform vaccine strain selection.

**FIGURE:**
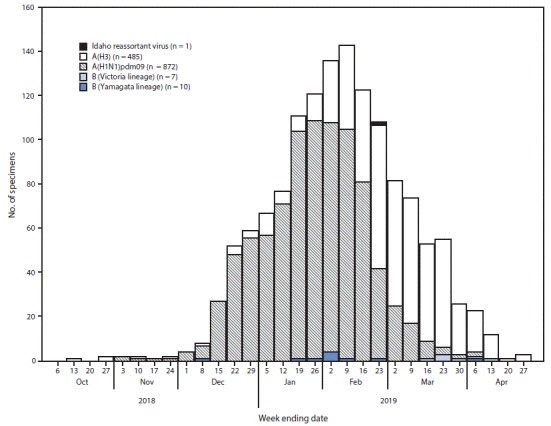
Number of respiratory specimens testing positive for influenza reported by Idaho Bureau of Laboratories, by influenza virus type,* subtype/lineage, and surveillance week (N = 1,375) — Idaho, October 6, 2018–April 27, 2019 * Illness onset date of Idaho reassortant infection was February 16, 2019.

## Footnotes

* https://www.who.int/influenza/vaccines/virus/recommendations/2018_19_north/en.

† https://www.cdc.gov/flu/weekly/overview.htm.

§ This new reassortant virus was identified and investigated in March 2019. Despite the delay in presenting the results of this investigation, the recommendation for continued surveillance of emerging influenza viruses has particular relevance at this time.
